# Mitochondria-Targeted Antioxidants as a Therapeutic Strategy for Chronic Obstructive Pulmonary Disease

**DOI:** 10.3390/antiox12040973

**Published:** 2023-04-21

**Authors:** Lauren H. Fairley, Shatarupa Das, Vivek Dharwal, Nadia Amorim, Karl J. Hegarty, Ridhima Wadhwa, Guntipally Mounika, Philip M. Hansbro

**Affiliations:** 1 Centre for Inflammation, School of Life Sciences, Faculty of Science, Centenary Institute and University of Technology Sydney, Sydney, NSW 2050, Australia; 2 Discipline of Pharmacy, Graduate School of Health, Faculty of Health, University of Technology Sydney, Ultimo, NSW 2007, Australia

**Keywords:** antioxidants, mitochondria, COPD, lung, ROS, pulmonary disease

## Abstract

Oxidative stress is a major hallmark of COPD, contributing to inflammatory signaling, corticosteroid resistance, DNA damage, and accelerated lung aging and cellular senescence. Evidence suggests that oxidative damage is not solely due to exogenous exposure to inhaled irritants, but also endogenous sources of oxidants in the form of reactive oxygen species (ROS). Mitochondria, the major producers of ROS, exhibit impaired structure and function in COPD, resulting in reduced oxidative capacity and excessive ROS production. Antioxidants have been shown to protect against ROS-induced oxidative damage in COPD, by reducing ROS levels, reducing inflammation, and protecting against the development of emphysema. However, currently available antioxidants are not routinely used in the management of COPD, suggesting the need for more effective antioxidant agents. In recent years, a number of mitochondria-targeted antioxidant (MTA) compounds have been developed that are capable of crossing the mitochondria lipid bilayer, offering a more targeted approach to reducing ROS at its source. In particular, MTAs have been shown to illicit greater protective effects compared to non-targeted, cellular antioxidants by further reducing apoptosis and offering greater protection against mtDNA damage, suggesting they are promising therapeutic agents for the treatment of COPD. Here, we review evidence for the therapeutic potential of MTAs as a treatment for chronic lung disease and discuss current challenges and future directions.

## 1. Introduction

Chronic obstructive pulmonary disease (COPD) is the third most common cause of death globally [1], with no effective cure. COPD is characterized by the limitation of airflow in the lungs, which leads to shortness of breath in addition to wheezing, chest tightness, and ongoing chronic cough [1]. Common pathologies of chronic airway inflammation, such as bronchitis, airway remodeling, collagen deposition, fibrosis, and mucus hypersecretion and/or emphysema, underly the condition [2,3]. Chronic exposure to cigarette smoke (CS) is the primary cause of COPD, but other causes include long-term exposure to lung irritants—such as air pollution, chemical fumes, or dust [4,5]. In addition, genetic risk factors have been identified in COPD, including α-1-antitrypsin deficiency (AATD), telomeropathies, and several other rare variants, and groups of common variants likely affect COPD heterogeneity [6,7,8].

Current treatments available for COPD include the use of bronchodilators [9], inhalation of corticosteroids [10], oxygen therapy, lung transplantation, or reduction surgeries, which mitigate disease symptoms and help to prevent exacerbations [11,12]. However, there are no currently available treatments that inhibit the progression or reverse the features of disease [13], and hence there is an urgent need for novel therapies that can prevent the progression of COPD. Although cessation of smoking is highly recommended for patients at all stages of COPD [14], disease progression fails to halt in the absence of exogenous stressors, suggesting that additional endogenous factors may underlie disease pathogenesis [15].

Oxidative stress has been identified as a key mechanism driving the pathogenesis of COPD, and mitochondria play an important role in these mechanisms through the generation of endogenous reactive oxygen species (ROS) [15,16]. Emerging evidence suggests that mitochondrial ROS (mtROS) contributes to widespread mitochondrial dysfunction in COPD and drives chronic inflammation in the lungs. Thus, therapeutic treatments aimed at targeting mtROS generation hold great promise for COPD. In this review, we discuss the role of mitochondria-derived oxidative stress in COPD and the therapeutic efficacy of MTAs as a treatment for chronic lung disease. Current challenges and new tools for targeting mtROS are discussed.

## 2. Oxidative Stress in COPD

Oxidative stress is a key pathological feature of COPD, with numerous studies reporting increased levels of oxidative stress markers such as ethane [17], malondialdehyde [18], hydrogen peroxide [19,20], and 8-isoprostane [21] in the breath of COPD patients. Oxidative stress refers to an imbalance in the production and accumulation of reactive molecules and free radicals derived from molecular oxygen, collectively referred to as reactive oxygen species (ROS). ROS arises from two sources in COPD; (1) exogenous ROS, which is caused by chronic inhalation of CS, as well as other environmental factors such as carbonyls/aldehydes, CO, NO_2_, SO_2_ etc. [5,22], and (2) endogenous ROS, which predominantly arises from damaged or dysfunctional mitochondria. Numerous studies have implicated endogenous ROS as a causative feature of COPD, due to the fact that oxidative stress fails to resolve in the absence of exogenous sources of ROS [23]. In line with this, oxidative stress markers remain elevated in COPD patients who are ex-smokers and never smokers [15,24], suggesting that endogenous ROS production may drive chronic deterioration of the lungs.

Mitochondria are the main energy producers of the cell and are also known to be one of the major sources of ROS (mtROS) [25]. MtROS are generated as by-products of the electron transport chain (ETC) located on the inner mitochondrial membrane during oxidative phosphorylation (OXPHOS) [26]. Electron leak from complex I and complex III of the ETC lead to a partial reduction in oxygen to form superoxide (O^−^). The sequential reduction in oxygen through the addition of electrons leads to the formation of several types of ROS including superoxide (O_2_^−^), hydrogen peroxide (H_2_O_2_), hydroxyl radical (OH), hydroxyl ion (OH^−^), and nitric oxide (NO) [27,28], leading to protein, lipid, and DNA damage.

Although normal levels of ROS play a crucial role in maintaining homeostatic processes, such as autophagy, pathogen killing, and resolution of inflammation [23,29,30,31], excessive ROS production causes damage to various cellular structures and has been shown to exacerbate COPD progression. For instance, ROS can lead to extracellular matrix (ECM) and blood vessel remodeling, mucus hypersecretion, as well as apoptosis [32,33] and senescence [34,35]. ROS have also been shown to inactivate growth factors in COPD, such as transforming growth factor beta (TGFβ), thus increasing fibrosis and activating matrix metalloproteinases (MMP) [36]. Conversely, mtROS inhibition has been shown to reduce airway hyperresponsiveness (AHR) and lung inflammation in mouse models of COPD [25,37], suggesting it plays a central role in the disease pathogenesis.

Recent studies have implicated mtROS production as important regulators of immune processes [38]. In line with this, endogenous ROS in the airways is primarily released from inflammatory cells such as macrophages, neutrophils, as well as epithelial and endothelial cells. Cigarette smoke (CS) triggers alveolar macrophages to produce ROS and subsequently releases mediators that attracts neutrophils and other inflammatory cells in the lungs [15,39]. ROS has been suggested to initiate a cascade of inflammatory responses in the lungs via activation of transcription factors such as nuclear factor (NF)-κB and activator protein (AP)-1, along with other signal transduction pathways including mitogen-activated protein kinases (MAPK) and phospoinositide-3-kinase (PI3K), resulting in increased pro-inflammatory factors [40,41,42,43]. In addition, oxidative stress in the lungs regulates nuclear histone modification such as methylation, acetylation, and phosphorylation that is suggested to cause chromatin remodeling, recruitment of basal transcription factors, and RNA polymerase II, leading to increased pro-inflammatory responses [40,41,42]. Studies have reported that CS-induced mtROS in COPD patients contributes to an alteration in mitochondrial fission and fusion proteins [44,45,46], increased oxidative stress gene signatures [47], lower mitochondrial membrane potential and ATP levels [48], impaired mitophagy and concomitant aggregation of damaged and dysfunctional mitochondria [35,49,50], and necrosis [51,52,53]. In line with this, immune cells in COPD exhibit altered metabolic function, including glycolysis and fatty acid oxidation [54,55,56,57,58], which are important processes required for ATP production to fuel energy demanding immune functions [59]. Subsequently, immune cells in COPD exhibit impaired immune functions, including phagocytosis, altered viral response, and increased cytokine production [60,61,62], suggesting a self-perpetuating cycle in which ROS exposure both initiates and maintains mitochondrial and immune cell dysfunction in COPD, contributing to widespread destruction of the airways.

The primary mechanism of ROS elimination is via endogenous antioxidant defense mechanisms, including antioxidant enzymes such as superoxide dismutases and catalase, and via direct antioxidants such as vitamin E, and glutathione (GSH). These antioxidants play a crucial role in reducing mtROS levels, by preserving the oxide/reduction equilibrium in the cell. However, the excessive generation of ROS in COPD leads to an imbalance between the rate of their formation and the antioxidant capacity. For instance, GSH is reduced in COPD patients and smokers [63,64,65], and correlates with disease severity [66]. Genetic studies have identified an association between extracellular superoxide dismutase (ECSOD) polymorphisms and the risk of developing COPD [67]. Furthermore, transcription factors FOXO3a and NRF2 are reduced in COPD patients, leading to downregulation of antioxidant genes [68,69]. Although some studies have reported increased antioxidant levels in smokers [70], these levels were unable to prevent the development of COPD [71], suggesting that these levels are either insufficient or easily overwhelmed by excessive ROS production [72]. Thus, therapeutic strategies aimed at enhancing antioxidant production and capacity are of great interest in COPD.

## 3. Non-Targeted Antioxidants in COPD

Due to the detrimental role that ROS plays in COPD pathogenesis, there is widespread interest in the use of conventional (non-targeted) antioxidants as a strategy for reducing oxidative stress in COPD. These include thiol-based antioxidant compounds (*N*-acetylcysteine (NAC) and carbocisteine), dietary antioxidants (vitamin C, vitamin E, resveratrol, and flavonoids), NADPH oxidase (NOX)inhibitors, and other small molecule antioxidants (reviewed in [15,73]). Indeed, non-targeted antioxidants have been shown to reduce inflammation, reduce oxidative stress, and attenuate cigarette smoke-induced changes in lung function in animal models of COPD and cells isolated from COPD patients [74,75,76]. However, despite the beneficial effects of non-targeted antioxidants in pre-clinical models of COPD, clinical trials in patients with COPD have produced contradictory results, and few have made it into clinical practice. For instance, whereas several small studies of NAC treatment in COPD patients reported reduced exacerbations, a larger clinical trial in 523 patients with COPD reported no reduction in exacerbations or disease progression following NAC treatment [77]. Two other thiol-based compounds, carbocisteine and erdosteine, have shown modest effects in reducing exacerbations in COPD patients [78,79,80]. However, these reductions only applied to mild exacerbations (but not moderate or severe), and benefits were only observed in conjunction with other treatments [81]. Studies investigating dietary supplementation with antioxidants have reported correlation and association with lung function parameters (reviewed by [82]), but largely reported no benefits on symptoms, lung function, or hospitalization for COPD [83]. Furthermore, dietary antioxidant supplementation has been linked to adverse effects and in some cases increases the risk of lung disease. For instance, studies have reported that lung cancer incidence was increased following β-carotene [84,85,86] and retinol supplementation [87]. Given the common environmental and genetic risk factors underlying both lung cancer and COPD [88], these findings suggest that these supplements may also exert harmful effects in COPD. This may be due to the possibility that dietary antioxidant supplementation may interfere with the absorption, transport, and metabolism of carotenoids and other micronutrients, thus increasing the risk of lung cancer [87,88]. In particular, higher concentrations of antioxidants have been suggested to be detrimental (reviewed in [89]). High-dose antioxidant exposure may interfere with the normal physiological roles of ROS required for tissue homeostasis, including autophagy, pathogen killing, and resolution of inflammation [23,29,30], or induce detrimental compensatory mechanisms, such as upregulation of mitogen-activated protein kinase (MAPK) pathways [90]. Overall, non-targeted antioxidants show only modest benefits in COPD patients, with no effect on lung function or disease progression, and high-dose supplementation may be detrimental. Current use of non-targeted antioxidants in COPD is hampered by their limited specificity in targeting ROS at its source, lack of knowledge concerning the optimal dose, and possible interference with physiologic processes, thus limiting their use in clinical settings.

## 4. Mitochondria-Targeted Antioxidants in COPD

Given that non-targeted antioxidants have largely failed to produce clinically relevant benefits in COPD patients, research is ongoing to develop novel mitochondria-targeted antioxidants (MTAs). These antioxidants are conjugated with a carrier, such as lipophilic cations, liposomes, or peptides, which enable the targeted delivery of bioactive ingredients into the mitochondria [91]. This mitochondria-specific transport enables the accumulation of high concentrations of antioxidants at the source of ROS generation, thus allowing for optimized dosing and specificity. Broadly, MTAs can be divided into four groups that are as follows: lipophilic cation-linked MTAs; liposome-encapsulated MTAs; peptide-based MTAs; and Manganese (Mn) Porphyrin-based MTAs [91] (Table 1). In this section, we will discuss currently available MTAs and their therapeutic potential for patients with COPD.

### 4.1. Lipophilic Cation-Linked MTAs

The mitochondrial membrane potential is generated by mitochondrial complexes in the inner mitochondrial membrane (IMM) and is required for oxidative phosphorylation and ATP generation. In general, mitochondria maintain a strong negative potential, approximately −180 mv, which makes the inner mitochondrial membrane impermeable to the passive diffusion of compounds [92]. Interestingly, this physical property of the IMM has been utilized to develop different lipophilic cation-linked MTAs [93]. A lipophilic cation such as triphenyl phosphonium (TPP) is conjugated with the selected target antioxidant. TPP, being lipophilic and positively charged, quickly passes through the lipid bilayer and is preferentially accumulated in the negatively charged mitochondrial matrix [94]. To date, multiple lipophilic cation-linked MTAs have been developed. The following are the most studied.

#### 4.1.1. MitoQ: MitoQ Is a Conjugation of TPP and Ubiquinone

Mitochondrial ubiquinone is a respiratory chain component in the lipid core of the IMM. It accepts two electrons from complexes I or II and is reduced to ubiquinol [95]. Multiple studies have shown that the ubiquinone pool is depleted with aging and under diseased conditions, and supplementation with ubiquinone improves mitochondrial function, reduces free radical production, and decreases lipid peroxidation [96,97,98]. However, unconjugated ubiquinone has low water solubility making its cellular uptake slow. Conjugating TPP leads to rapid accumulation of ubiquinone in mitochondria [99], thus reducing mitochondrial oxidative stress.

Several in vitro studies have revealed protective effects of MitoQ using cells from COPD patients, or in response to cigarette smoke extract (CSE) exposure. MitoQ has been found to reduce CSE-induced ROS levels and attenuate autophagy in human umbilical vein endothelial cells (HUVECs) [100] and Beas-2B cells [52], potentially via inhibiting PINK1 stabilization and consequent DRP1 phosphorylation [52]. MitoQ restored endothelial barrier integrity, as well as decreased inflammation by the NF-κB and NLRP3 inflammasome pathways in endothelial cells [100]. Both MitoQ and Tiron attenuated the proliferation of airway smooth muscle (ASM) cells from human patients with COPD, and Tiron further attenuated cytokine secretion in these cells [25].

**Figure 1:**
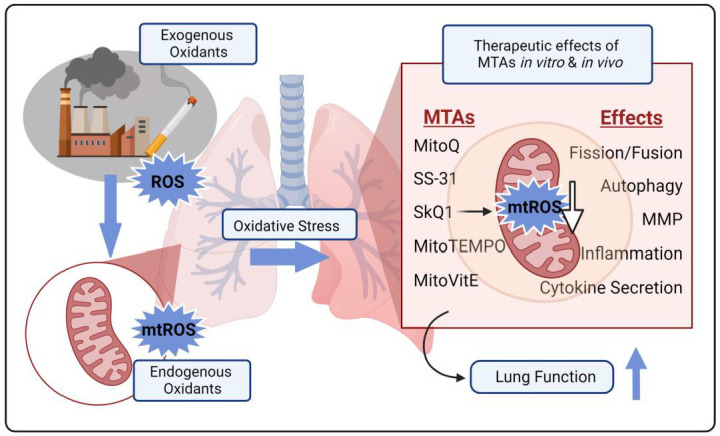
Therapeutic effects of mitochondria-targeted antioxidants in COPD. Endogenous exposure to oxidants triggers endogenous mtROS production in COPD. In vitro studies using cells from COPD patients, or in response to cigarette smoke (CS) exposure, have reported protective effects on mitochondrial ROS levels, fission and fusion, mitophagy, MMP, as well as inflammation and cytokine secretion. Abbreviations: ROS: reactive oxygen species; mtROS: mitochondrial ROS; MMP: mitochondrial membrane potential.

In line with these findings, pre-clinical studies using animal models of COPD have reported therapeutic effects of MTAs in vivo. For instance, the treatment of mice with MitoQ reversed airway hyperresponsiveness (AHR), reduced total BAL cell counts, restored mitochondrial membrane potential, and reduced ROS levels in an ozone-induced model of COPD [25]. Another recent study showed that treatment of mice with MitoTEMPO reduced lung inflammation scores, attenuated inflammatory cytokine levels, and reduced serum 8-OHdG and mtROS levels in ozone-exposed mice [37]. In addition, this same study showed that MitoTEMPO treatment inhibited the expression of mitochondrial complex II and IV in lung tissue, and also inhibited the expression of mitochondrial fission/fusion-related proteins DRP1 and MFF, as well as NLRP3. However, no effect of MitoTEMPO on lung function was observed. Taken together, promising data from both in vitro and in vivo studies suggest that MTA exert beneficial effects in COPD, and the translational potentials of MTAs in clinical trials warrants further investigation (Figure 1).

MitoQ is currently the only commercially available MTA (available as a dietary supplement) and has shown significant success in Phase II clinical trials for patients with hypertension [101], Parkinson’s [102], and liver disease [103]. In addition, MitoQ has been shown to improve motor function [104] and vascular function in healthy older adults [105]. A 2019 study by Kwon and colleagues reported protective effects of MitoQ on measures of vascular function and hyperemic response to both single and continuous passive leg movement in COPD patients [106], suggesting that MitoQ is a promising approach to combat cardiovascular disease in patients with COPD. However, no effects on exacerbations or lung function were reported. Two clinical studies investigating the effects of MitoQ in COPD patients are currently in progress [107,108], and there are additional ongoing clinical studies investigating MitoQ in other lung diseases, including asthma (NCT04026711), cystic fibrosis (NCT02690064), COVID-19 (NCT05373043, NCT05381454), and respiratory viral infections (NCT05381454) (Table 2). The results of these studies will be highly informative about the potential therapeutic efficacy of MTAs in COPD moving forward. One of the biggest challenges in clinical trials using MitoQ is establishing the optimal drug dose and standardizing treatment methodology. Thus, in current and ongoing clinical studies of MitoQ in COPD, establishing standardized dosing will be of significant clinical importance.

#### 4.1.2. SkQ1 and SKQR1

SkQs are a subclass of MTAs consisting of plastoquinone, an electron carrier and antioxidant, conjugated with TPP to obtain SkQ1 or its analog plastoquinonyl decylrhodamine 19 (SkQR1). Studies have shown that plastoquinone may exert improved antioxidant effects compared to ubiquinone. SkQ1 exerts antioxidant effects at lower concentrations than MitoQ in vitro [112]. Further, the “window” between anti- and pro-oxidant concentrations of SkQ1 and SkQR1 is much larger than MitoQ, thus reducing the risk of off-targeted effects [113]. Treatment with SkQ1 significantly ameliorated defective phagocytosis in monocyte-derived macrophages (MDMs) generated from COPD patients [114]. A phase 2 clinical trial has already been conducted with SKQ1 against Keratoconjunctivitis Sicca. The study shows that SkQ1 treatment improves the functional state of the cornea by reducing dry eye symptoms such as dryness, burning, grittiness, and blurred vision [115,116]. There are currently no clinical trials on SkQ1 or SKQR1 in COPD.

#### 4.1.3. MitoTEMPOL and MitoTEMPO

MitoTEMPO and MitoTEMPOL are the piperidine nitroxides 4-hydroxy-2,2,6,6-tetramethylpiperidine-1-oxy (TEMPOL) and 2,2,6,6-tetramethylpiperidine-1-oxy (TEMPO) linked to TPP [117]. In vitro studies have shown that these compounds are interconverted into the nitroxide and the oxoammonium forms and act as superoxide dismutase mimic [118]. Growing reports suggest that treatment with MitoTEMPO or MitoTEMPOL reduces free radical production, lipid peroxidation, oxidative stress, and inflammation in a wide range of different disorders [119,120,121]. MitoTEMPO has been shown to exert protective effects in vitro by reducing CSE-induced ROS levels and attenuating mitochondrial fragmentation in HBECs [45] and human pulmonary artery smooth muscle cells [122], as well as reducing neutrophil extracellular traps (NETs) formation in polymorphonuclear neutrophils isolated from the peripheral blood of COPD patients [123].

#### 4.1.4. MitoVitE

MitoVitE is a conjugate of vitamin E (Vit-E) attached to a TPP cation [124]. Vit-E, or α-tocopherol, is a potent antioxidant in the IMM. The phenolic group of the chromanol ring present in vit-E donates hydrogen to the free peroxyl and alkoxyl radicals generated during lipid peroxidation and oxidative phosphorylation, thus acting as a free radical scavenger [125,126]. The antioxidant and anti-inflammatory potential of MitoVitE has been reported in preclinical studies in different disorders, including pneumonia-related sepsis [127], and neuropathic pain [128], but no studies have yet been conducted in COPD.

### 4.2. Peptide-Based MTAs

Mitochondria-targeted peptides, also known as SS (Szeto–Schiller) peptides, are novel antioxidants that target mitochondria-associated oxidative stress. Structurally, they consist of alternating aromatic residues and basic amino acids (aromatic-cationic peptides). The tyrosine or dimethyl-tyrosine residues present in these peptides are responsible for free radical scavenging properties. SS-01 (H-Tyr-D-Arg-Phe-Lys-NH_2_), 2. SS-02 (H-Dmt-D-Arg-Phe-Lys-NH_2_), SS20 (H-Phe-D-Arg-Phe-Lys-NH_2_), and SS31 (H-D-Arg-Dmt-Lys-Phe-NH_2_) are the different types of SS peptides that are generated, and SS31 is most studied among them [129]. The main advantages of SS peptides over the TPP-like MTAs are that their cellular uptake is energy independent, and their uptake in mitochondria is independent of MMP [130]. A large body of evidence shows that SS31 treatment reduces ROS production, improves mitochondrial functioning, and prevents mitochondrial structural changes and phospholipid oxidation in different disorders [131,132,133,134]. SS-31 has also been shown to be safely tolerated and exert protective effects in phase I clinical trials of patients with heart failure [135] and phase II trials of reperfusion injury patients [136]. Despite this, there are currently no clinical trials on peptide-based MTAs in COPD patients.

### 4.3. Mn (III) Porphyrin-Based MTAs

Cationic Mn (III) N-substituted pyridyl porphyrins are superoxide dismutase mimics known to alleviate the superoxide stress. MnTE-2-PyP5^+^, MnTnHex-2-PyP5^+^, and MnTnBuOE-2-PyP5^+^ are the most important Mn (III) Porphyrins (MnPs) that have been studied. Studies show that the positive charge and the lipophilic alkyl chains direct the preferential accumulation of these compounds in mitochondria [137]. In addition to their role as free radical scavengers, MnPs are known to modulate the activity of transcription factors such as nuclear factor κB, nuclear factor E2-related factor 2, and hypoxia-inducible factor [138,139,140]. Clinical trials are already ongoing utilizing MnTnBuOE-2-PyP5^+^ against Squamous Cell Carcinoma and Anal Cancer (NCT03386500).

### 4.4. Liposome-Encapsulated MTAs

Liposomes are lipid bilayer membrane vesicles used as nanocarriers for drug or bioactive substance delivery to cells. The advantage of liposome encapsulation is that it does not alter the activity or structure of the bioactive substance to be delivered. Liposome-encapsulated MTAs enter the cells through micropinocytosis. Following entry through macropinosomes disruption, mitochondrial membrane fusion occurs, and the antioxidant is delivered to the mitochondrial matrix [92,141]. So far, different antioxidants such as quercetin, resveratrol, curcumin etc., have been liposome encapsulated and tested against various pathological conditions [142,143,144], and improved therapeutic effects of encapsulated antioxidants compared to non-encapsulated ones have been reported [145,146]. However, to date, no studies have tested the therapeutic efficacy of liposome-encapsulated MTAs in COPD.

## 5. Non-Targeted versus Mitochondria-Targeted Antioxidants

In general, MTAs are thought to confer greater protection against oxidative stress compared to untargeted antioxidants. For instance, Kolosova and colleagues compared the effects of the MTA SkQ1 with NAC on markers of aging in senescence-accelerated OXYS rats and found that SkQ1 prevented age-related decline to a greater degree compared to NAC, despite the higher dose of NAC used [141]. Similarly, Jauslin and colleagues reported that MitoQ and MitoVitE were several hundredfold more potent in protecting Friedreich Ataxia fibroblasts against oxidative stress compared to the untargeted antioxidants Trolox and idebenone [147]. Consistent with this, Oyewole and colleagues reported that MitoQ and Tiron elicited greater protection against UVA and H_2_O_2_-induced mitochondrial DNA (mtDNA) damage in human dermal fibroblasts, compared to nontargeted antioxidants (resveratrol, curcumin, and N-acetyl cysteine) [148]. The reason for the superior effects of MTAs over nontargeted antioxidants has largely been attributed to their efficient pharmacokinetics, better absorption rate, and specific targeting of ROS at its source, thus allowing for more optimal dosing and less non-specific effects [91,145,149].

## 6. Limitations and Future Perspectives for MTAs in COPD

Despite the promising results from MTA use in pre-clinical models of COPD, these compounds exhibit several limitations, which may hamper their translational potential. For instance, the method by which mitochondria are targeted by MTAs is important as it can produce potential off target effects. TPP, being lipophilic and positively charged, quickly passes through the lipid bilayer and is preferentially accumulated in the negatively charged mitochondrial matrix [94]. However, the TPP moiety of MTAs has been shown to inhibit oxidative phosphorylation independent of antioxidant effects [146], and both the TPP-conjugated MTAs MitoQ and SkQ1 were reported to impair mitochondrial function in vitro [94,150]. It should be noted that the concentrations of MTAs used in these studies were significantly larger than those associated with beneficial effects in vivo, and thus their potential effects in disease models are unclear. Since the TPP-conjugated MTAs MitoQ and SKQ1 can also impair mitochondrial function at higher concentrations, it is important to understand their dose-response effects.

Paradoxically, these types of carriers, which otherwise are essential to facilitate MTA accumulation inside the mitochondria, can also impair their uptake in damaged cells. Targeting of the mitochondria using lipophilic cations requires the presence of an intact MMP for mitochondrial localization [151]. However, it is well established that MMP is reduced in a range of cell types in COPD [152,153]. This can result in MTAs selectively targeting healthy cells where the MMP is intact instead of cells involved in the aberrant production of mtROS. This could destabilize mtROS homeostasis required for healthy cell function and have unintended detrimental effects.

To prevent the adverse effects of the TPP-conjugated MTAs, newer approaches in targeting mitochondria have been investigated that do not rely on exploiting the mitochondrial potential. For instance, peptide-based MTAs such as SS-31 have been developed, using Szeto–Schiller (SS) tetrapeptides, whose cellular uptake is energy independent and does not rely on MMP [130]. Nonetheless, as the TPP-based compound MitoQ is the most widely studied MTA and is currently the only compound being tested in clinical trials of COPD, further studies are needed to compare the safety and efficacy of SS-31 with other MTAs, such as MitoQ. In addition, alternate approaches to targeting mitochondria have been suggested, such as liposome-encapsulated MTAs and polymer-based nanocarriers and nanoparticles [149,154,155].

Another potential limitation of MTAs that has thus far not been studied in the context of COPD is their interference with physiological ROS functions. A commonly reported issue with nontargeted antioxidant use is that they may also impair the homeostatic functions of ROS, such as autophagy, pathogen killing, and resolution of inflammation [23,29,30,31]. Furthermore, heterogenous ROS are observed in COPD, and specific ROS subtypes may play a larger role in detrimental disease features. Thus, the generation of novel MTAs directed at targeting specific detrimental ROS subpopulations is a promising avenue for drug development.

MTAs are often dispersed throughout the body, despite the fact that oxidative damage is predominantly localized to the lung in COPD. Consequently, concentrations of MTAs in the lung of COPD patients may be inadequate, and optimal dosing may be difficult to achieve. Again, nanocarriers may present a promising way to combat these limitations, as MTAs can be loaded into nanosized drug carriers that can be engineered to selectively accumulate in specific disease sites/tissues [156].

Finally, obtaining conclusive evidence from clinical trials in COPD has been historically limited by inconsistent methodology. As outlined previously, there are a range of known causes of COPD that produce different phenotypes of disease [4], and often studies exhibit differences in treatment doses, administration, and disease stage. Future studies and trials should attempt to minimize uncertainty by addressing these issues and targeting cohorts with similar COPD phenotypes. Overall, pre-clinical data using animal and cellular models suggest that MTAs exert beneficial effects in COPD, and MitoQ has been shown to be safely tolerated and improves vascular dysfunction in COPD patients. However, clinical studies testing MTAs are still in phase 1, and thus further testing is needed to ascertain the clinical significance of these compounds for COPD.

## 7. Conclusions

MTAs have shown promise as effective therapeutics in in vitro and ex vivo models as well as pre-clinical animal models of COPD and warrant further investigation in clinical trials. Currently, human clinical trials on MTAs in COPD and other chronic lung diseases are still ongoing and their findings will provide critical information on the future use of MTAs as a treatment for COPD. Alongside these trials, various issues should be addressed, including a better understanding of the most relevant oxidation pathways in COPD, standardized dosing, and the mechanistic role of MTAs in physiologic ROS processes. Thus, to fully capitalize on the potential therapeutic benefits of MTAs, more translational research in human patients with COPD is required to test the feasibility of transitioning from bench to bedside. 

## Figures and Tables

**Table 1:** Mitochondria-targeted antioxidants and their mode of action.

Type	Mode of Action
Lipophilic cation-linked MTAs	Different antioxidants such as Ubiquinone, plastoquinone, piperidine nitroxides, and α-tocopherol are linked to lipophilic cations such as TPP. The positive charge of lipophilic cations results in the preferential accumulation of these antioxidants in mitochondria.
Peptide-Based MTAs	These are small, positively charged peptides with alternating aromatic residues and basic amino acids. The tyrosine or dimethyl-tyrosine residues present in these peptides are responsible for free radical scavenging properties.
Mn (III) Porphyrin-based MTAs	These are superoxide dismutase mimics.
Liposome-encapsulated MTAs	Different antioxidants such as quercetin, resveratrol, curcumin, etc. are encapsulated in liposomes. This results in increased cellular uptake through micropinocytosis and mitochondrial transfer through membrane fusion.

**Table 2:** Mitochondria-targeted antioxidants in clinical trials for chronic lung disease.

Intervention	Condition	Effects	Status	Phase	ClinicalTrial.gov Identifier	Ref
MitoQ	COPD	N/A	Ongoing	N/A	NCT05605548	[107]
MitoQ	COPD	N/A	Ongoing	1	NCT02966665	[108]
MitoQ	COPD	Enhanced FMD Enhanced PLM and LBF	Completed			[106]
MitoQ	Asthma	N/A	Ongoing	1	NCT04026711	[109]
MitoQ	CF	N/A	Ongoing	N/A	NCT02690064	[110]
Mito-Q + Exercise	COVID-19	N/A	Ongoing	N/A	NCT05373043	[111]
MitoQ	COVID-19	N/A	Ongoing	1/2	NCT05381454	[110]
MitoQ	Respiratory Viral Infections	N/A	Ongoing	1/2	NCT05381454	[110]

Abbreviations: FMD: flow-mediated vasodilation; PLM: passive leg movement; LBF: leg blood flow; CF: cystic fibrosis.

## Data Availability

Not applicable.
